# Supplementary figures and images for: Toxoplasma IMC1 is a central component of the subpellicular network and plays critical roles in parasite morphology, replication, and infectivity
Source: PLoS Pathog. 2026 May 11;22(5):e1014080. doi: 10.1371/journal.ppat.1014080 (PMC13175489; doi:10.1371/journal.ppat.1014080)

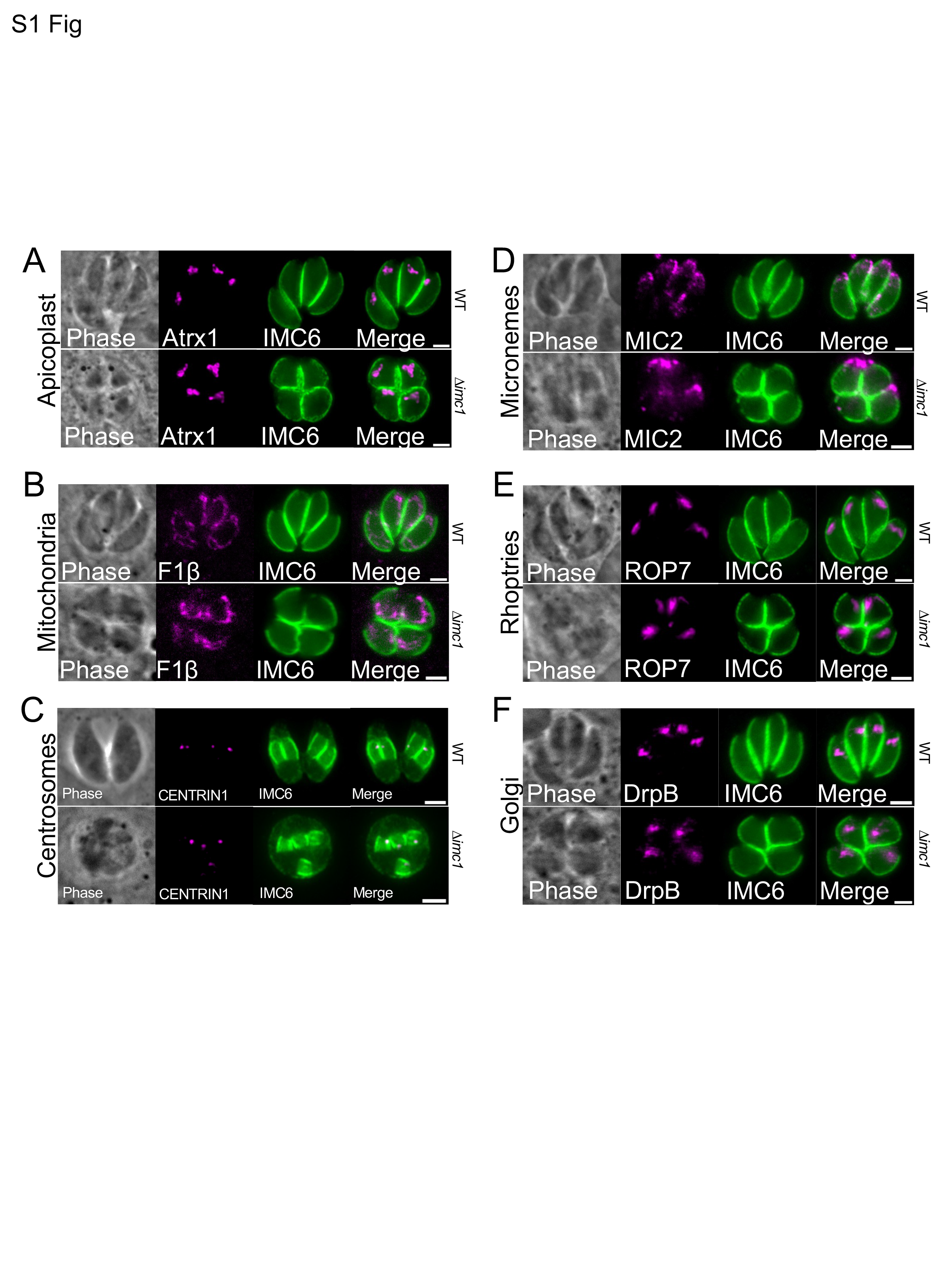

Supplement: S1 Fig — (A-F) IFAs of wild-type and Δimc1 parasites, showing normal morphology of the indicated organelles. (A) The apicoplast was detected with anti-ATrx1 (magenta). (B) Mitochondria were detected with anti-F1β (magenta). (C) The centrosome was detected with anti-CENTRIN1 (magenta). (D) Micronemes were detected with anti-MIC2 (magenta). (E) Rhoptries were detected with anti-ROP7 (magenta). (F) The Golgi (trans-Golgi) was detected with anti-DrpB (magenta). All IFAs were costained with anti-IMC6 (green). All scale bars are 2 µm. (TIF) [file ppat.1014080.s001.tif]

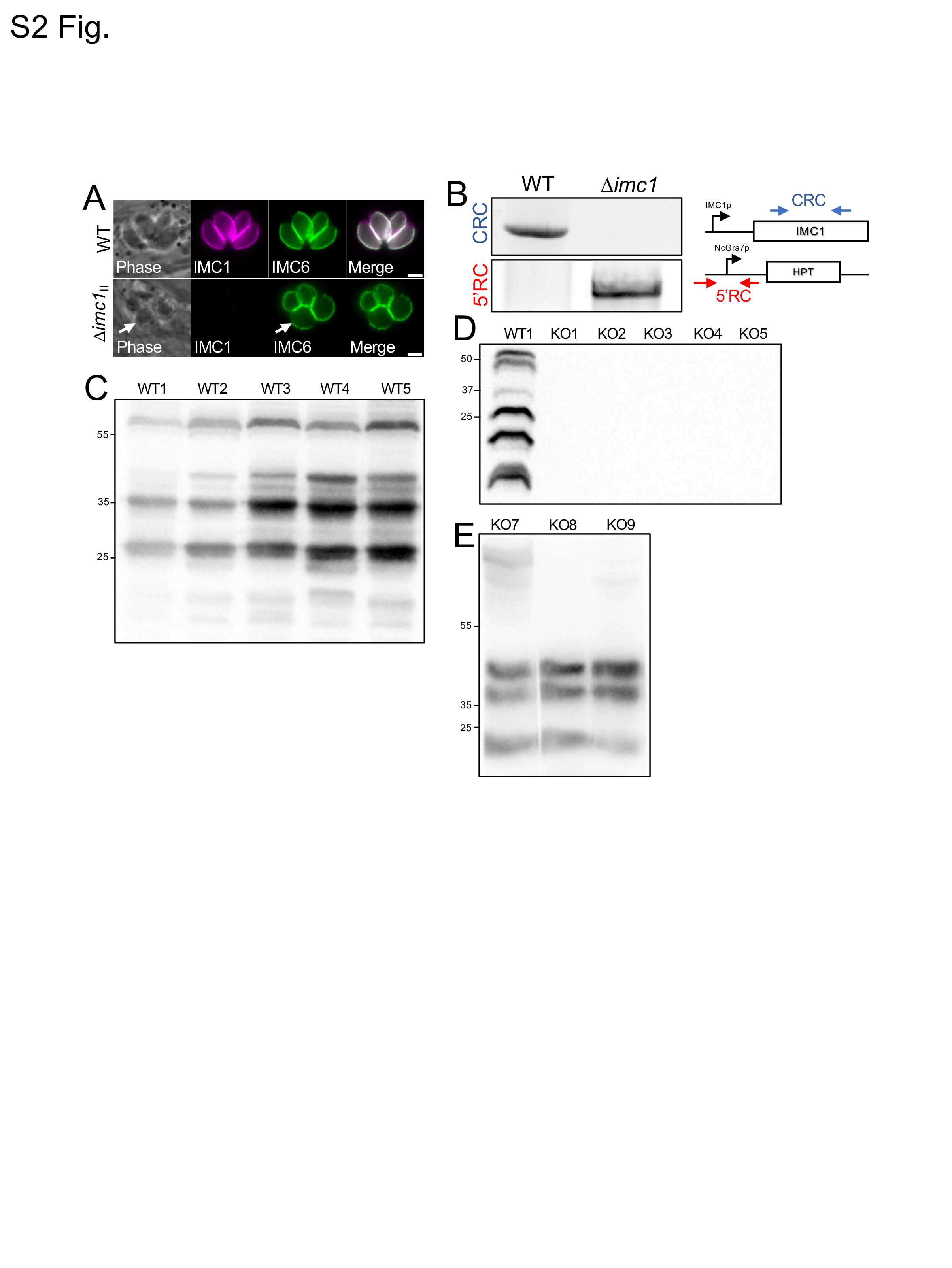

Supplement: S2 Fig — A) IFA of intracellular WT parasites showing proper localization of IMC1 (top). IFA of intracellular Δimc1II parasites showing absence of IMC1 and swollen morphology (bottom, arrows). B) PCR verification of the genomic loci from WT (Pru∆hxgprt∆ku80) and ∆imc1II parasites. Diagram demonstrates the primers used to amplify regions of the IMC1 coding sequence (blue arrows, coding region check) and the regions containing the 5’ site of recombination for the knockout locus (5’ recombination check, red arrows). C, D) Western blots of whole parasite lysates probed with infection sera at 30 days post infection showing that the mice fail to seroconvert with a ten-fold increase (114 vs. 1235 pfu) in infectious dose of the ∆imc1II strain compared to the wild-type infected mice which all seroconvert. E) Seroconversion is obtained at a high dose (~200,000 pfu) of the ∆imc1II strain. All scale bars are 2 µm. IFA, immunofluorescence assay; WT, wild-type; KO, ∆imc1II; CRC, Coding Region Check; 5’RC, 5’ Recombination Check. (TIF) [file ppat.1014080.s002.tif]

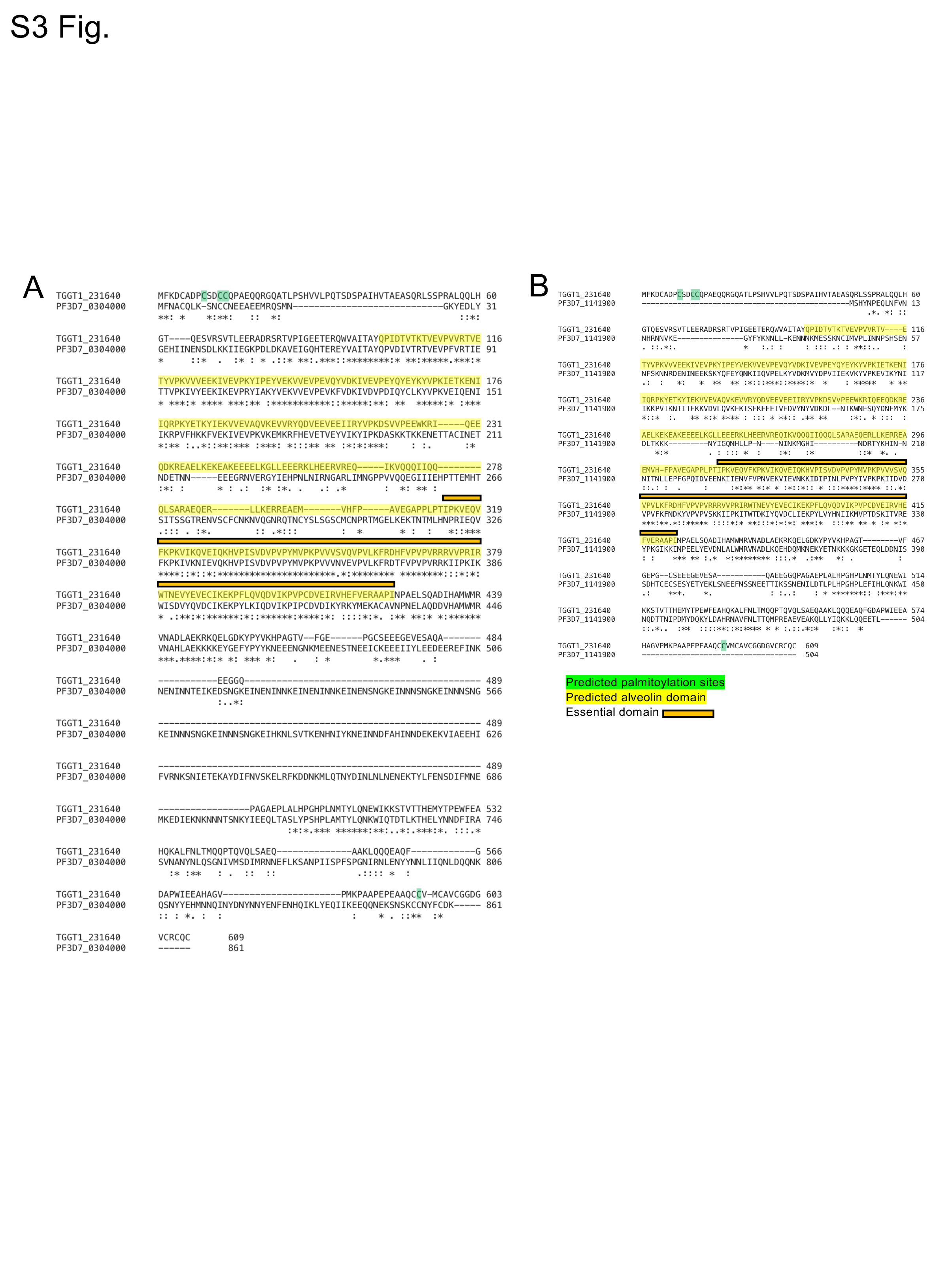

Supplement: S3 Fig — A, B) Alignment of Toxoplasma IMC1 (TgGT1_231640) with IMC1a (panel A, PF3D7_0304000) or IMC1b (panel B, PF3D7_1141900) from Plasmodium falciparum. For T. gondii, the predicted palmitoylation sites are highlighted in green, the alveolin domain is highlighted in yellow, and the region that is essential for IMC targeting is highlighted with an orange box. Asterisks indicate identity, a colon indicates conservation between groups of strongly similar properties, and a period indicates conservation between groups of weakly similar properties. (TIF) [file ppat.1014080.s003.tif]

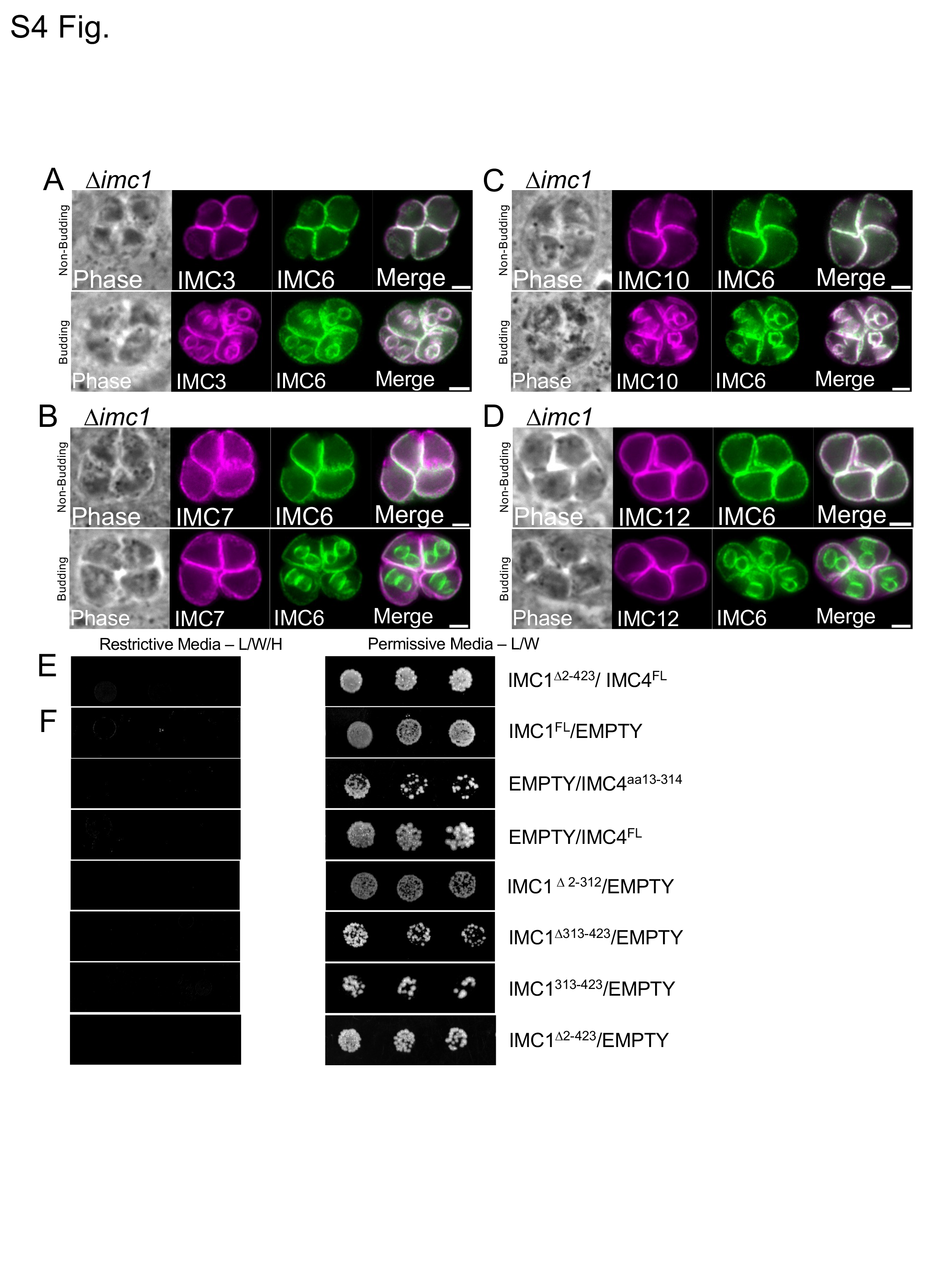

Supplement: S4 Fig — A-D) IFAs showing that IMC3, IMC7, IMC10, and IMC12 are unaffected in both budding and non-budding ∆imc1 parasites. The proteins are detected with their respective antibodies and IMC6, also unaffected, is used for costaining. E) Images of yeast spot assay showing no binding between IMC1∆2-423 and IMC4FL. F) Images of controls for yeast two-hybrid experiments with empty vector partners showing a lack of autoactivation by failure to grow on restrictive media (L/W/H). Growth on permissive media (L/W) is also shown. All scale bars are 2 µm. (TIF) [file ppat.1014080.s004.tif]

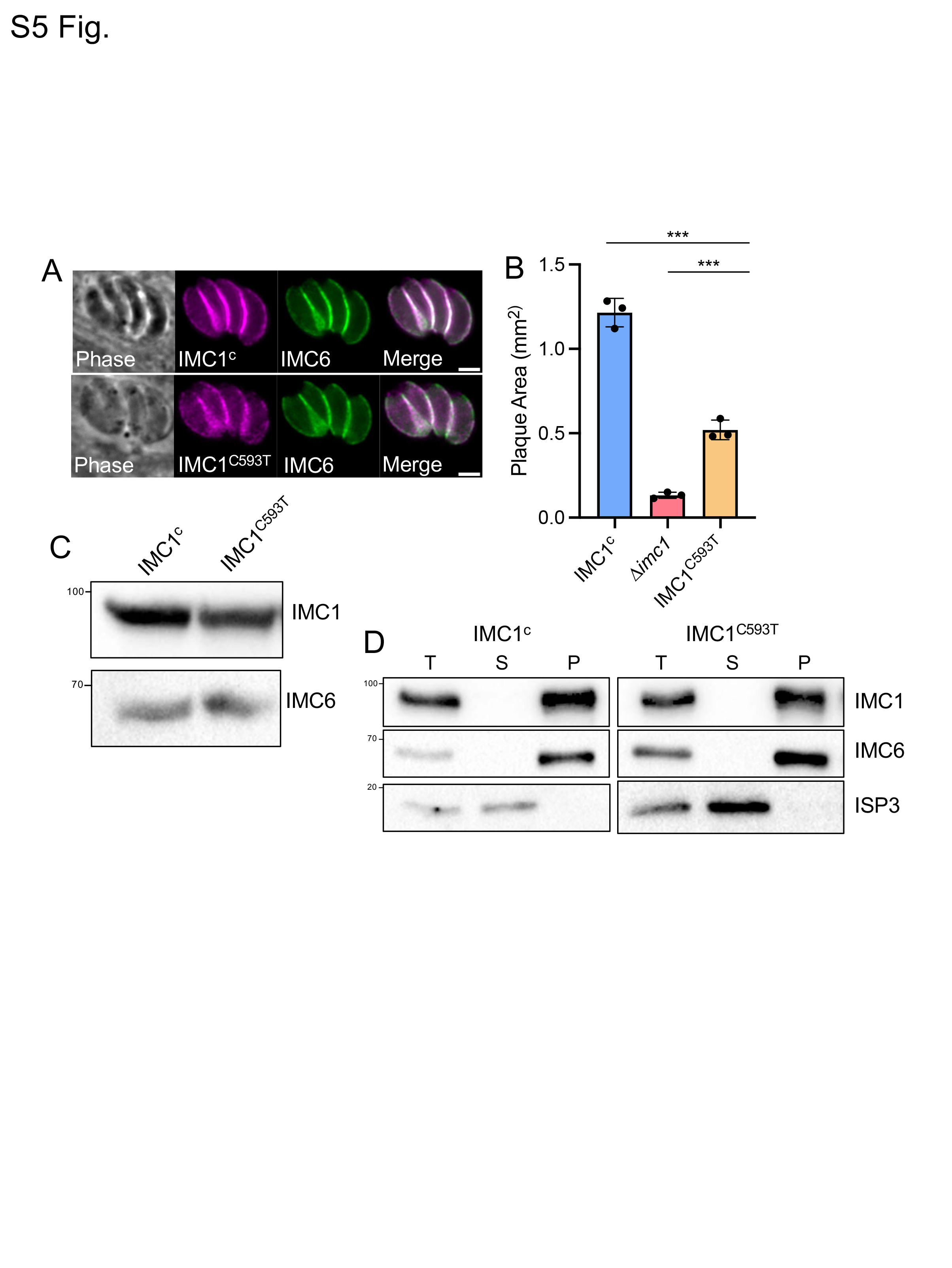

Supplement: S5 Fig — A) IFA showing IMC1C593T localizes to the IMC as shown by colocalization with IMC6. Green, anti-IMC1; Magenta anti-IMC6. B) Quantification of plaque assays showing that IMC1C593T only partially rescues the ∆imc1 strain. C) Western blots of IMC1c and IMC1C593T parasites showing similar levels of the complemented proteins. IMC6 is used as a loading control. D) Detergent fractionation of IMC1c and IMC1C593T demonstrating that both proteins partition to the detergent insoluble pellet. IMC6 and ISP3 are controls for the detergent insoluble fraction and soluble fraction, respectively. *** P = 0.0003, 0.0004. Statistical significance was determined using two-tailed t tests. T, total cell lysate. S, detergent soluble fraction. P, detergent insoluble fraction. All scale bars are 2 µm. (TIF) [file ppat.1014080.s005.tif]
